# Ground Level PM_2.5_ Estimates over China Using Satellite-Based Geographically Weighted Regression (GWR) Models Are Improved by Including NO_2_ and Enhanced Vegetation Index (EVI)

**DOI:** 10.3390/ijerph13121215

**Published:** 2016-12-07

**Authors:** Tianhao Zhang, Wei Gong, Wei Wang, Yuxi Ji, Zhongmin Zhu, Yusi Huang

**Affiliations:** 1 State Key Laboratory of Information Engineering in Surveying, Mapping, and Remote Sensing, Wuhan University, Wuhan 430079, China; tianhaozhang@whu.edu.cn (T.Z.); jiyuxi_ss@163.com (Y.J.); zhongmin.zhu@whu.edu.cn (Z.Z.); mavis_huang@whu.edu.cn (Y.H.); 2 Collaborative Innovation Center for Geospatial Technology, Wuhan 430079, China; 3 College of Information Science and Engineering, Wuchang Shouyi University, Wuhan 430064, China

**Keywords:** nationwide ambient PM_2.5_, MODIS (Moderate Resolution Imaging Spectroradiometer) AOD, satellite-derived NO_2_ column density, enhanced vegetation index, geographically weighted regression

## Abstract

Highly accurate data on the spatial distribution of ambient fine particulate matter (<2.5 μm: PM_2.5_) is currently quite limited in China. By introducing NO_2_ and Enhanced Vegetation Index (EVI) into the Geographically Weighted Regression (GWR) model, a newly developed GWR model combined with a fused Aerosol Optical Depth (AOD) product and meteorological parameters could explain approximately 87% of the variability in the corresponding PM_2.5_ mass concentrations. There existed obvious increase in the estimation accuracy against the original GWR model without NO_2_ and EVI, where cross-validation R^2^ increased from 0.77 to 0.87. Both models tended to overestimate when measurement is low and underestimate when high, where the exact boundary value depended greatly on the dependent variable. There was still severe PM_2.5_ pollution in many residential areas until 2015; however, policy-driven energy conservation and emission reduction not only reduced the severity of PM_2.5_ pollution but also its spatial range, to a certain extent, from 2014 to 2015. The accuracy of satellite-derived PM_2.5_ still has limitations for regions with insufficient ground monitoring stations and desert areas. Generally, the use of NO_2_ and EVI in GWR models could more effectively estimate PM_2.5_ at the national scale than previous GWR models. The results in this study could provide a reasonable reference for assessing health impacts, and could be used to examine the effectiveness of emission control strategies under implementation in China.

## 1. Introduction

Numerous previous studies reported that atmospheric particulate matter emitted from both anthropogenic and natural sources exert influences on climate change and environmental deterioration [1,2]. Many epidemiological studies have shown that exposure to fine suspended particles with aerodynamic diameter less than 2.5 μm (PM_2.5_) are linked with cardiovascular and respiratory diseases [3,4,5,6]. With vast consumption of energy and rapid economic development, China has suffered from severe PM_2.5_ pollution and the related social problems have caused wide concerns [7,8]. Although an air quality monitoring network has been established in China since 2013, large-scale estimation of PM_2.5_ is not practical due to the limited spatial coverage of the monitoring stations [9]. Consequently, there exists an urgent need to acquire a spatially resolved characterization of human exposure to PM_2.5_ at the national scale in China [10].

In order to make up for the blanks in ground measurements, methods using remote sensing by satellite have been adopted to estimate ground-level PM_2.5_ mass concentrations [11,12]. There exists a direct relationship between the atmospheric particles and satellite-derived Aerosol Optical Depth (AOD) because AOD represents the quantity of light removed from a beam by the role of aerosol scattering or absorption during its path [13,14,15,16]. Previous studies proposed establishing empirical models to correlate ground-level PM_2.5_ and satellite derived AOD (e.g., linear, nonlinear, and logarithmic models) [13,17,18,19]. Superior models have been established for better predicting the PM_2.5_ concentrations combined with meteorological parameters or based on atmospheric transport models, such as the mixed effects model (MEM) [20], the artificial neural network model (ANN) [17,21,22], and the chemical transport model (CTM) [23]. Since the correlation between AOD and PM_2.5_ should vary along with the spatial context due to different geographical areas possessing different aerosol types [9,24,25], the Geographically Weighted Regression (GWR) model, which better constrains the spatial variability in a large-region regression, has been adopted to estimate geographical elements in large regions [26,27]. The particulate matter vertical distribution has been additionally taken into consideration from a physics perspective, which could improve the correlation between PM_2.5_ and AOD [28]. Moreover, previous study of the GWR model combined with physical corrections indicated that the atmospheric vertical feature could be embedded in the GWR model via Planetary Boundary Layer Height (PBLH) for better estimation accuracy of atmospheric particulates at the national scale [29]. Nevertheless, these statistical models rely greatly on observation from satellites and meteorological parameters, and anthropogenic emissions need additionally consideration, such as traffic density [30].

In this study, the molar concentration of OMI-NO_2_ (Ozone Measuring Instrument) and MODIS-EVI (Moderate Resolution Imaging Spectroradiometer) were introduced into the GWR model, considering that these two variables can be measured via satellites at a large spatial scale. A different GWR model was then established to predict PM_2.5_ concentrations at the national scale using fused MODIS-AOD based on a dark target and deep blue algorithm, meteorological parameters, daily tropospheric NO_2_ molar concentration, and fixed 16-day composite EVI. For quantitatively evaluating the model performance, a leave-one-out cross-validation was adopted to demonstrate the relationship between measured PM_2.5_ and estimated PM_2.5_, and results from the previous GWR model without NO_2_ and EVI were also provided for contrast. Moreover, the two-year annual spatial distribution of the satellite-derived PM_2.5_ at the national scale was demonstrated, analyzed, and discussed.

## 2. Materials and Methods

### 2.1. Ground PM_2.5_ Measurements

**Figure 1:**
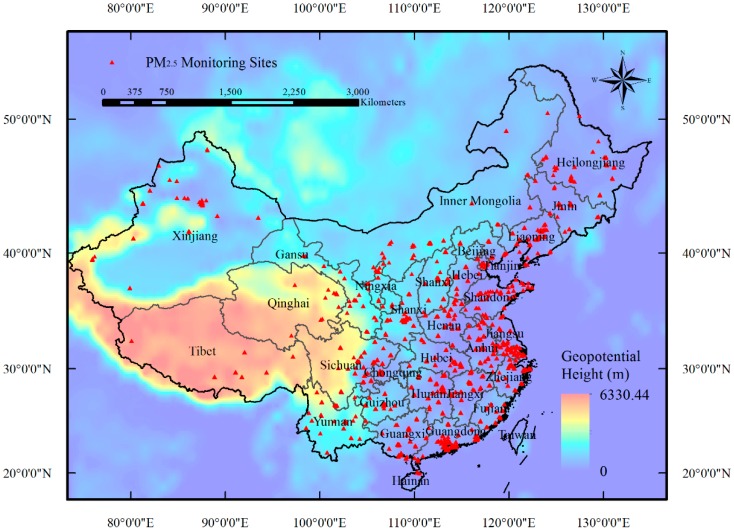
Spatial distribution of PM_2.5_ monitoring stations (solid red dots) from which data were gathered in this study.

Hourly ground-level PM_2.5_ measurements in China from 1 January 2014 to 31 December 2015 were collected primarily from the official website of the China Environmental Monitoring Center (CEMC) [31] As demonstrated in Figure 1, more than 1300 air quality monitoring stations have been built up covering residential cities in all provinces of China by the end of 2014. According to the officially released documents of the Chinese Ministry of Environmental Protection (MEP), the PM_2.5_ data were measured using the tapered element oscillating microbalance method (TEOM) or the beta-attenuation method, combined with periodic calibration [32].

### 2.2. Moderate Resolution Imaging Spectroradiometer (MODIS) Aerosol Products

The MODIS aboard the NASA Earth Observing System (EOS) satellite, Aqua, was shown to provide aerosol products with assured quality when compared with other satellite sensors [33,34,35,36]. In this study, we use the Collection 6 (C6) aerosol products because these products have generally been proven to attain fine accuracy by validation against ground monitoring observations of sun photometers from the Aerosol Robotic Network (AERONET) in China [37,38]. The Aqua AOD datasets used in this study, which were retrieved based on the relationship between the radiation value of specific bands over land and the aerosol optical depth, were distributed in Hierarchical Data Format (HDF) format from the NASA Goddard Space Flight Center [39] at the national scale (longitude (73°40’–135°2.5’ E), latitude (3°52’–53°33’ N)). The second generation Deep Blue (DB) was expanded to cover brighter desert/urban areas and vegetated land surfaces, which preferably makes up for deficiencies in the Dark Target (DT) algorithms [40,41]. However, the spatial resolution of the Aqua Level 2 AOD using DB algorithms is 10 × 10 km, while the spatial resolution of the Aqua Level 2 AOD using DT algorithms reaches 3 × 3 km. Thus, the AOD datasets were fused by 3 km DT AOD and 10 km DB AOD, based on the concept of complementary advantages, in order to make merging AOD possess advantages on both spatial resolution and spatial coverage [42]. Moreover, only retrievals reaching the required quality assurance (QA) were used (corresponding to flag QA = 3 for DT; flag QA = 2 or QA = 3 for DB), ensuring the accuracy of the fused AOD.

### 2.3. Aerological and Surface Meteorological Parameters

Aerological parameters, including PBLH, and surface meteorological parameters, consisting of surface relative humidity, u-components, and v-components of surface winds, surface temperature, and surface atmospheric pressure, were collected from the National Centers for Environmental Prediction (NCEP) reanalysis datasets with 1° spatial resolution. Atmospheric product and land surface data taken every six hours are available in the NCEP datasets, which are available on its website [43]. 

### 2.4. Satellite-Derived EVI and NO_2_ Data

The Enhanced Vegetation Index (EVI), which is calculated by surface reflectance in near-infrared, red, and blue bands, is an optimized vegetation index to represent vegetation biomass. The EVI, instead of the normalized difference vegetation index (NDVI) data, was adopted in this study to simply characterize the reduction effect by vegetation on PM_2.5_ concentrations because the EVI is more sensitive to variations in regions having high biomass, which is an obvious advantage over NDVI [44]. Although the calculation of EVI is similar to NDVI, the distortions caused by atmospheric particles in the reflected light have been corrected, and the EVI product barely becomes saturated when scanning regions containing large amounts of chlorophyll [45]. EVI data with 1 km spatial resolution and 16-day temporal resolution was collected from the NASA LAADS website [39].

The daily tropospheric column density of NO_2_ were collected from the NASA OMI level 2 nitrogen dioxide dataset with 0.25° spatial resolution [46]. The OMI NO_2_ algorithm could compute accurate vertical column densities from NO_2_ slant column densities, retrieved by spectral fitting. Since NO_2_ is a short-life trace gas and has a tight relation to anthropogenic emissions and energy consumption [47,48,49,50], we utilized the satellite-derived NO_2_ molar concentration as a simplified proxy of anthropogenic emissions.

### 2.5. Data Integration

From the perspective of time, the Aqua MODIS and Aura-OMI passes the equator at approximately 1:30 p.m. local time, thus all the aerological and surface meteorological variables were correspondingly selected around 2:00 p.m. local time (corresponding to UTC = 6:00 a.m.). Moreover, the daily NO_2_ dataset and 16-day EVI dataset were respectively utilized to represent the daily extent of anthropogenic emissions and vegetation coverage for 16 days. In addition, a Kriging resampling approach was applied to ensure spatial consistency of all independent variables, which were demonstrated in Table 1 in detail. Moreover, the meteorological parameters and AOD values were both selected from the pixel in which the ground-level monitor is located.

### 2.6. Model Development, Comparison, and Validation

Because previous research has shown that the relationship between AOD and PM_2.5_ obviously varies according to the spatial context, and the correlation coefficients could lead to poor accuracy of estimation when using global parameters [36]. To solve this problem, a GWR model has been established in this study, and the adaptive Gaussian bandwidth search method was utilized to accommodate the uneven distribution of ground-monitoring stations. The structure of the GWR model developed in this study is expressed in the following equation:

(1) $$PM_{2.5,l,d}=\beta_{0,l,d}+\beta_{1,l,d}Revised_{AOD_{l,d}}+\beta_{2,l,d}ST_{l,d}+\beta_{3,l,d}RH_{l,d}+\beta_{4,l,d}PS_{l,d}+\beta_{5,l,d}WS_{l,d}+\beta_{6,l,d}NO_{2,l,d}+\beta_{7,l,d}EVI_{l,d}$$

where the *PM*_2.5*,l,d*_ (μg/m^3^) is the ground-level PM_2.5_ concentration at location *l* on day *d*; *β*_0*,l,d*_ denotes the intercept at location *l* on day *d*; *β*_0*,l,d*_ to *β*_7*,l,d*_ represent location-specific slopes; *Revised_AOD_l,d_* (no unit) is the Aqua-MODIS AOD fused products revised by PBLH (m) at location *l* on day *d*. As demonstrated in previous studies, physics corrections based on vertical distribution could make remarkable improvement in the relationship between AOD and PM [29,51]. Because the ground monitoring stations measured mass concentration data of near ground PM_2.5_, optical parameters for the ambient atmosphere should be measured instead of satellite-derived AOD that characterizes the entire atmospheric column. *ST_l,d_*, *RH_l,d_*, *PS_l,d_* and *WS_l,d_* are the surface temperature (K), surface relative humidity (%), atmospheric pressure (Pa), and surface wind speed (m/s), respectively, at location *l* on day *d*. *NO*_2*,l,d*_ represents the column molar concentration of NO_2_ at location *l* on day *d*, and *EVI_l,d_* represents the enhanced vegetation index at location *l* on 16-day *d*.

Moreover, to examine the effectiveness of introducing NO_2_ and EVI into the GWR model as representatives of the anthropogenic emissions and the reduction effect by vegetation on PM_2.5_. As a contrast, a GWR model without parameters of NO_2_ and EVI was established using the same meteorological factors:

(2) $$PM_{2.5,l,d}=\beta_{0,l,d}+\beta_{1,l,d}Revised\_AOD_{l,d}+\beta_{2,l,d}ST_{l,d}+\beta_{3,l,d}RH_{l,d}+\beta_{4,l,d}PS_{l,d}+\beta_{5,l,d}WS_{l,d}$$

In addition, a 10-fold cross validation analysis [52] was conducted to validate the quality of the model by comparing the estimated PM_2.5_ against the monitoring values. The entire dataset was randomly split into 10 folds, with around ten percent of the total data in each subset, and the model was then fitted by nine folds with one fold set for validation in each cross-validation circle. This process was completely repeated 10 times so as to validate every fold. Furthermore, the estimation equation, decision coefficient R^2^, and mean absolute error (MAE, μg/m^3^) were calculated to evaluate the model performance.

## 3. Results and Discussion

### 3.1. Descriptive Statistics

**Figure 2:**
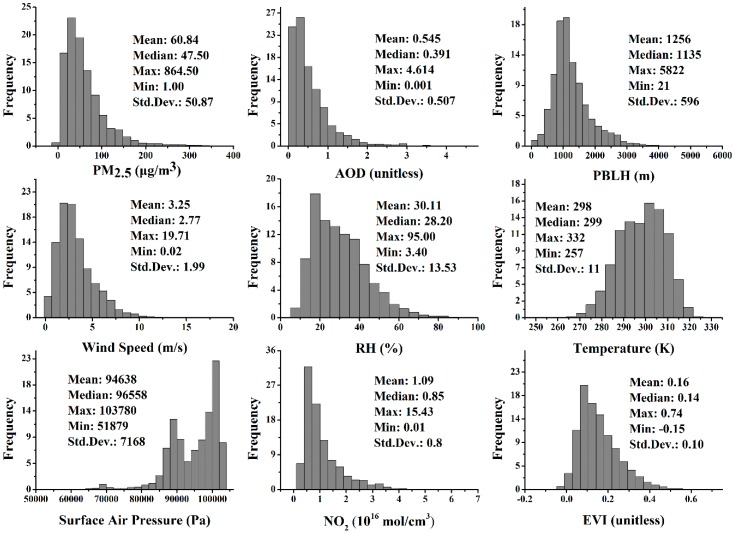
Histograms and descriptive statistics for PM_2.5_, AOD, Planetary Boundary Layer Height (PBLH), wind speed, surface relative humidity (RH), surface temperature, surface air pressure, NO_2_ column density, and EVI in the model fitting.

The histograms and descriptive statistics of all the variables in the GWR model are illustrated in Figure 2, including the dependent variable and independent variables. It demonstrates that, apart from the surface air pressure displaying a bimodal distribution, which is possibly caused by the obvious elevation difference in southwest China, the remaining variables were approximately log-normal distributed. Overall, the mass concentrations of PM_2.5_ ranged from 1 to 864.5 μg/m^3^ with an annual average of 50.38 μg/m^3^ and standard deviation (Std. Dev.) of 45.33 μg/m^3^. The AOD frequency histograms have a shape similar to the measured PM_2.5_ with an annual average AOD value of 497.49 and Std. Dev. of 470.72. Considering the Chinese standard of ambient air quality [53], the averaged mass concentration of PM_2.5_ ranging from 2014 to 2015 exceeded the level 2 standard (35 μg/m^3^).

### 3.2. Model Fitting, Validation, and Comparison

**Figure 3:**
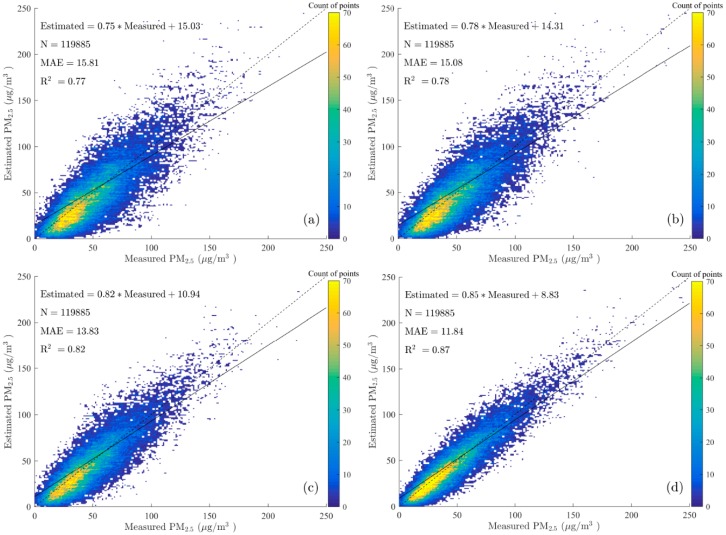
Scatter plot of model fitting and cross-validation for the GWR model: results of classical GWR model with vertical corrected AOD and meteorological parameters (**a**); results of GWR model only adding NO_2_ (**b**); results of GWR model only adding EVI (**c**); and results of new GWR model adding NO_2_ and EVI (**d**). The solid line and dotted line are the regression line, and y = x reference line, respectively.

The mass concentrations of PM_2.5_ and corresponding estimations were obtained across China from 1 January 2014 to 31 December 2015. The original GWR model, the compared GWR model only adding NO_2_, the compared GWR model only adding EVI, and newly developed GWR model were tested using the same datasets. There existed 119,885 pieces of data added to the model, and the cross-validation (CV) results for the GWR model are shown in Figure 3. The R^2^ of the CV validation increased from 0.77 to 0.78 when only adding NO_2_, with MAE decreased from 15.81 μg/m^3^ to 15.08 μg/m^3^. In addition, the R^2^ of the CV validation increased from 0.77 to 0.82 when only adding EVI, with MAE decreased from 15.81 μg/m^3^ to 13.83 μg/m^3^. It showed that the introduction of NO_2_ into the GWR model slightly improved the performance of model, while the introduction of EVI into the GWR model could substantially improve the performance of model. The R^2^ of the CV validation increased from 0.77 to 0.87 when adding two parameters, which means that both parameters are relevant to the new developed GWR model. This two-year estimation result could account for approximately 87% of the variability in the corresponding PM_2.5_ mass concentrations, which was relatively great at the national scale when comparing to other studies in China (cross validation R^2^ achieved 0.64 and 0.79 in annual average, respectively) [38,54]. Moreover, the MAE calculated from the new model decrease from 15.81 μg/m^3^ to 11.84 μg/m^3^ against the original model, indicating that PM_2.5_ estimated using the GWR model with NO_2_ and EVI agreed better with the measurements. Moreover, according to the linear regression results, the regression line (solid black) of the new model better fit the actual line (dotted black), which illustrates that the introduction of NO_2_ and EVI into GWR model could improve the estimation of PM_2.5_, since it could achieve higher R^2^ and better consistency with measurements.

However, the slope of the linear fitting equation was less than 1.0 and the intercept was positive, which suggests that the model is inclined to overestimate when the measurements of PM_2.5_ are less than around 60 μg/m^3^, and to underestimate when the measurements of PM_2.5_ are larger than approximately 60 μg/m^3^. Furthermore, the demarcation point, which was calculated by the point of intersection of linear fitting line and y = x reference line, was roughly equal in both GWR models. This phenomenon was probably caused by a mechanism of the GWR model that the coefficients of variables tend to be similar when they are geographically close to each other; therefore, the estimated values seem to be slightly averaged in a small region. Therefore, the particular value at an intersection point depends on the measured value of the dependent variable (PM_2.5_) to a large extent.

### 3.3. Annual Estimation of PM_2.5_ Mass Concentration

**Figure 4:**
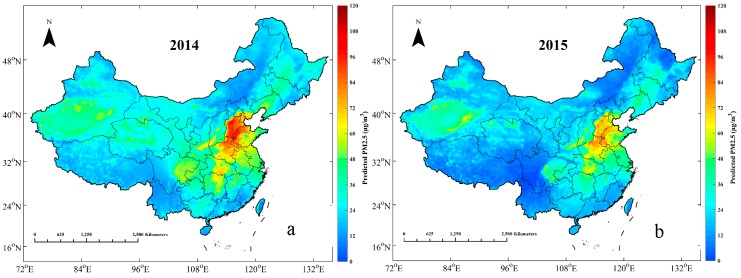
Estimations of annual averaged AOD-derived PM_2.5_ in 2014 (**a**) and 2015 (**b**) when corresponding dataset values were available.

The estimated annual averaged PM_2.5_ mass concentrations in 2014 and 2015 are illustrated in Figure 4. From a macro perspective, the spatial distributions of PM_2.5_ are generally consistent from the year 2014 to 2015. The highest values of PM_2.5_ were observed in the Jing-Jin-Ji Region (including Beijing, Tianjin, and Hebei), followed by Central China (including Hunan, Hubei, and Henan), and the Xinjiang Autonomous Region. In the Jing-Jin-Ji Region, the annual averaged PM_2.5_ mass concentrations were generally higher than 80 µg/m^3^ in 2014, and higher than 60 µg/m^3^ in 2015. High levels of industrialization and urbanization, as well as the activities of dense human populations, have led to severe PM_2.5_ pollution in these areas [55,56]. Based on the World Health Organization (WHO) Air Quality Interim Target (IT) levels, WHO IT-1 set the PM_2.5_ mass concentration standard of 35 μg/m^3^ [57], which means that most of regions in the North China Plain still suffer from severe fine particulate pollution. The situation is similar in central China, where most regions slightly exceeded the WHO IT-1 standard until 2015. The cleanest regions are in Tibet, Yunnan, and Hainan, where the annual average PM_2.5_ values are generally lower than 20 µg/m^3^.

Nevertheless, the policy-driven efforts in energy conservation and emission reduction should not be ignored. Although there still exists PM_2.5_ pollution in many residential areas, the apparent decrease in the overall trend is worth recognition. Not only the severity of PM_2.5_ pollution but also the spatial coverage of heavy PM_2.5_ pollution was alleviated, to a certain extent, from 2014 to 2015, especially in the North China Plain, Central Plain, scattered regions in the northeastern area and along the southern coast. 

Although the model was demonstrated to estimate the spatial distribution of PM_2.5_ effectively in most of areas in China, there still exist estimation errors in several regions. For instance, the mass concentrations of PM_2.5_ in the Xinjiang Autonomous Region should be relatively high because the Tarim Basin, which is located in the southern section of the Xinjiang Autonomous Region, is mostly covered by the Taklimakan Desert, where dust aerosols come mostly from primal generation and entrained effects across eastern Asia [58]. However, the prediction results demonstrated that the annual averaged mass concentration of PM_2.5_ in the Xinjiang Autonomous Region were less than 70 µg/m^3^, which was slightly below the measurements. The possible reason for this phenomenon in the model prediction is the introduction of NO_2_. As widely acknowledged, the Xinjiang Autonomous Region is mostly covered by desert, which leads to relatively lower density of NO_2_ measured by satellite. Thus, due to the positive fitted coefficients of NO_2_, the prediction results would be less than measurements when there was no ground air-quality monitoring station in the desert. Moreover, the predictions of PM_2.5_ in 2014 in Tibet, Qinghai, Gansu, northern Inner Mongolia, and western Sichuan were a little bit overestimated. This overestimation phenomenon primarily resulted from uneven distribution of the ground level PM_2.5_ monitoring sites in 2014. There was primary coverage in large urban centers and sparse coverage in rural areas, especially in the western regions of the country. Less coverage of ground level PM_2.5_ monitoring stations in these regions would directly result in the increase of bandwidth in the GWR model, further leading to the consequence that regional modeling would be influenced primarily by the farther surrounding areas. Since the GWR model adopts an adaptive bandwidth searching method, the bandwidth will increase when the ground stations are sparse, further decreasing the effects of model performance. However, along with construction of the Chinese air-quality monitoring network, the situation was alleviated in 2015, and this issue will gradually be resolved. In the current data conditions, the model could only achieve anticipative efficacy of estimation in eastern China.

## 4. Conclusions

The estimation of mass concentrations of PM_2.5_ at the national scale was conducted using a GWR model, by introducing NO_2_ and EVI, combined with fused Aqua MODIS AOD and meteorological parameters. According to the results of 10-fold cross-validation, the introduction of NO_2_ and EVI as independent variables in the GWR model obviously improved the quality of estimation, where the decision coefficient R^2^ increased from 0.77 to 0.87, and MAE decreased from 15.81 to 11.84 μg/m^3^ against the results from the original model. Moreover, the newly developed model and original model both tended to overestimate when the measured value of PM_2.5_ was less than around 60 μg/m^3^, but tended to underestimate when the measured value was larger than approximately 60 μg/m^3^. In addition, this particular value that marks this boundary, which was calculated from the intersection point of the linear fitting line and y = x as the reference line, depends on the measurements of dependent variable (PM_2.5_) to a large extent.

According to the spatial distribution of annual average PM_2.5_ mass concentrations in 2014 and 2015, the highest values of PM_2.5_ arose in the Jing-Jin-Ji Region, followed by Central China and the Xinjiang Autonomous Region. Furthermore, the NO_2_ embedded GWR model would be inclined to underestimate in the desert regions that possess relatively low density of NO_2_ measured by satellite. In addition, regional modeling would be influenced more by surrounding areas when the coverage of ground level monitoring stations is insufficient. Thus, in current data conditions, it is hard to achieve anticipative performance of estimation in western China. Nevertheless, this situation was alleviated in 2015 and will gradually be resolved following construction of the air-quality monitoring network in China.

In general, the introduction of NO_2_ and EVI into the GWR model could more effectively estimate PM_2.5_ at the national scale compared to the original GWR model using satellite-derived AOD and meteorological parameters. Further research will be conducted on the modeling algorithm and source apportionment of PM_2.5_ to achieve better performance. The estimation of PM_2.5_ mass concentrations at the national scale could provide a reasonable reference for assessing health impacts in China, and for examining the effectiveness of the emission control strategies under implementation.

## Figures and Tables

**Table 1:** Relevant Level 2 SDS titles and contents for Aqua MODIS AOD at 550 nm.

Data	Source	Temporal Resolution	Spatial Resolution	Spatial Resolution after Resampling
PM_2.5_	Ground-level Measurement	1 h	-	-
DT-AOD	Aqua-MODIS	1 day	3 km	3 km
DB-AOD	Aqua-MODIS	1 day	10 km	3 km
Meteorological Parameters	NCEP Reanalysis	6 h	100 km	3 km
NO_2_	Aura-OMI	1 day	25 km	3 km
EVI	Aqua-MODIS	16 days	1 km	3 km

DT: Dark Target; DB: Deep Blue; EVI: Enhanced Vegetation Index; AOD: Aerosol Optical Depth; MODIS: Moderate Resolution Imaging Spectroradiometer; NCEP: National Centers for Environmental Prediction; OMI: Ozone Measuring Instrument; SDS: Safety data sheet.
